# Supplementary material for: The contribution of endocytic mediators to itch transmission
Source: Front Mol Neurosci. 2026 Jun 29;19:1875249. doi: 10.3389/fnmol.2026.1875249 (PMC13358001; doi:10.3389/fnmol.2026.1875249)
Supplement: Supplementary file 1 [file Table_1.docx]

**Table S1.** siRNA sequences used to knockdown AAK1, Dnm1 and Dnm3. m, mouse.

| Target | Dharnmacon Sequence |
| --- | --- |
| *mAAK1* | GAAGGUGGAUUCGCUCUUG, GGACUCAAAUCUCCUGACA, GCAGAUAUUUGGGCUCUAG, AAAUGUGCCUUGAAACGUA. |
| *mDnm1* | GCGUGUACCCUGAGCGUGU, UGGUAUUGCUCCUGCGACA, GGGAGGAGAUGGAGCGAAU, GCUGAGACCGAUCGAGUCA. |
| *mDnm3* | CAACGAAGGCUGACGAUAA, GCUCAGAGUUCCUGCGAAA, GUGAAUGGAACUCGUAUAA, GCAGAAACAGACCGCGUAA |
| *Control* | UGGUUUACAUGUCGACUAA, UGGUUUACAUGUUGUGUGA, UGGUUUACAUGUUUUCUGA, UGGUUUACAUGUUUUCCUA. |
