# Supplementary figures and images for: The contribution of endocytic mediators to itch transmission
Source: Front Mol Neurosci. 2026 Jun 29;19:1875249. doi: 10.3389/fnmol.2026.1875249 (PMC13358001; doi:10.3389/fnmol.2026.1875249)

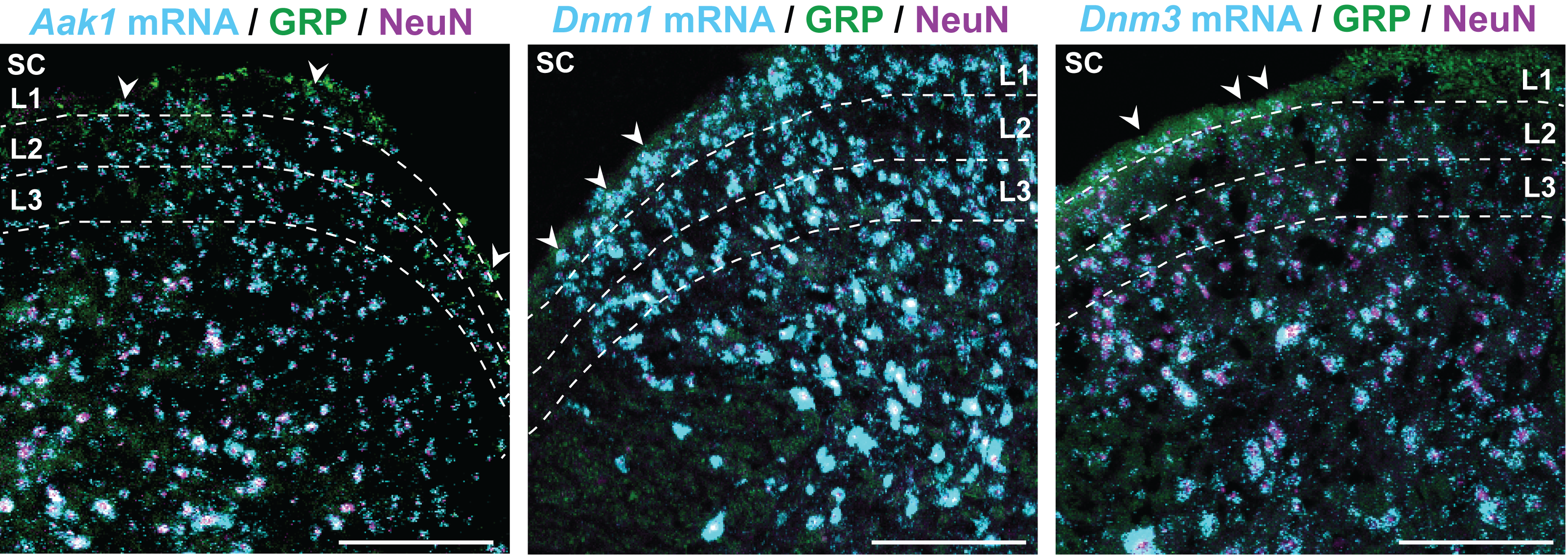

Supplement: Supplementary Figure 1 — Localization of Aak1, Dnm1, and Dnm3 mRNA in itch-related neurons in the dorsal horn of the spinal cord. Immunofluorescence detection of GRP and NeuN and RNAScope detection of Aak1, Dnm1,and Dnm3 mRNA in mouse spinal cord. Scale bar 50 μm. Arrows indicate mRNA expression within GRP neurons. Representative images. GRP, gastrin-releasing peptide; Aak1, adaptor-associated kinase 1; Dnm, dynamin. [file Image_1.TIF]

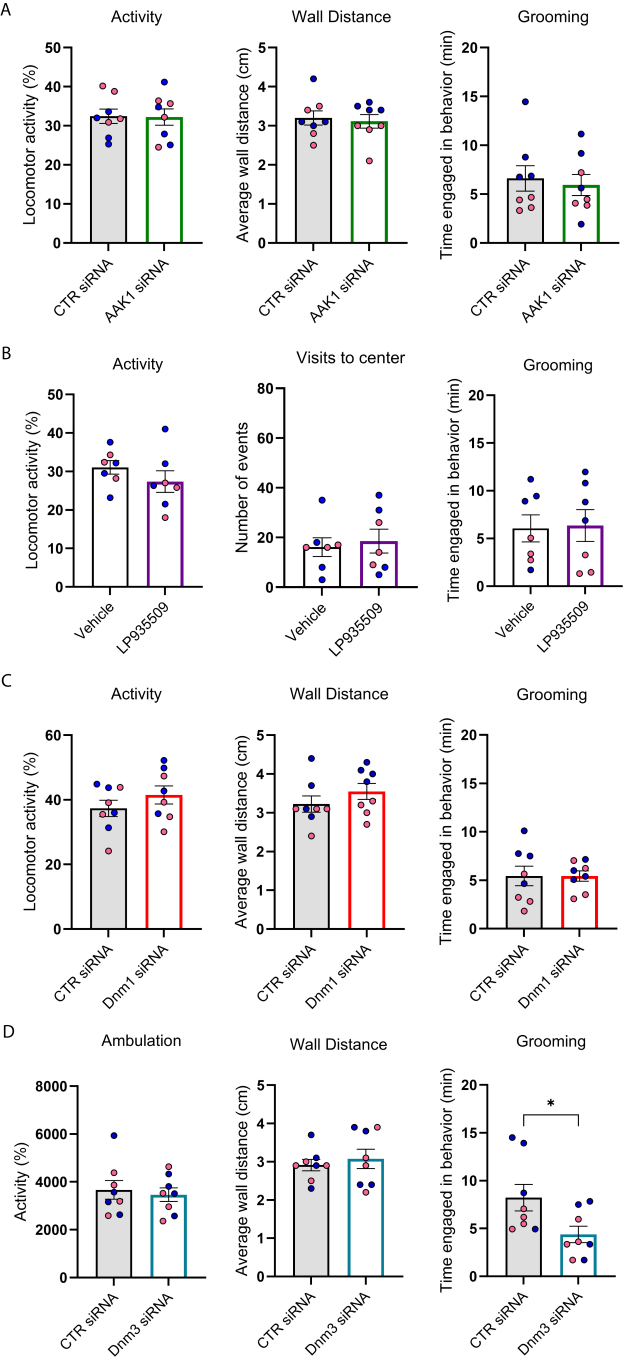

Supplement: Supplementary Figure 2 — Non-evoked behaviors after treatment. Non-evoked behavior in chloroquine-induced scratch recorded for 30 min at 2 days after intrathecal administration of AAK1 siRNA (A), LP965509 (10 μg/5 μL) (B), Dnm1 siRNA (C), Dnm3 siRNA or their respective controls (D). n = 8 mice per group (four male and four female mice), blue circles represent male and pink circles represent female mice. Data shown as mean ± SEM. *P < 0.05, vs. CTR siRNA group, parametric unpaired 2-tailed t-test. [file Image_2.TIF]

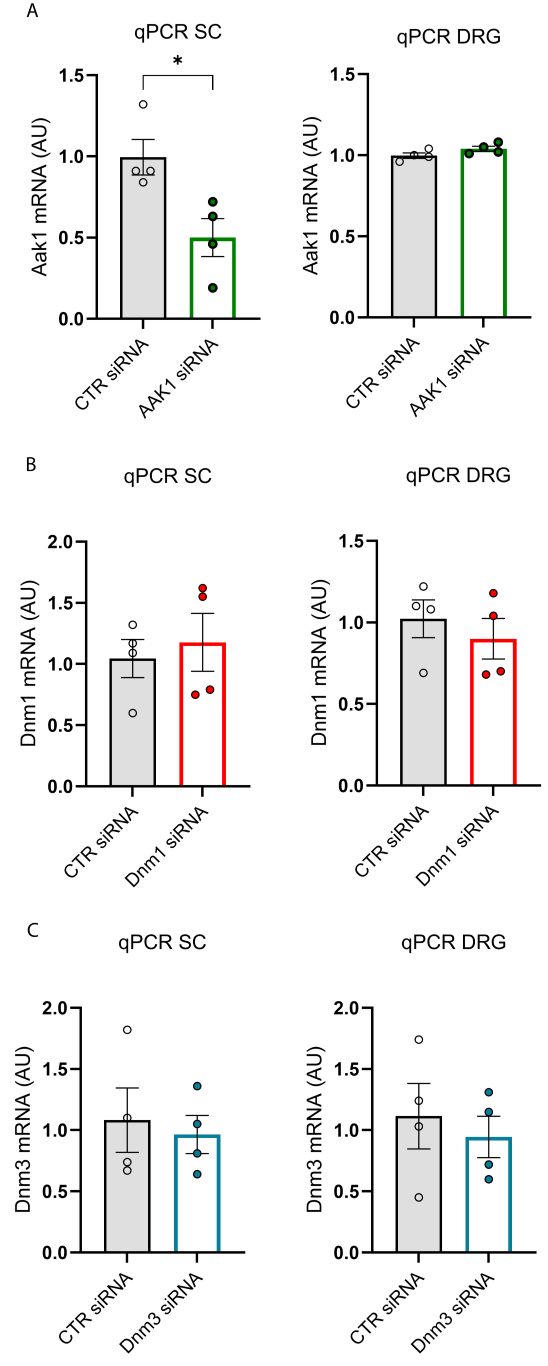

Supplement: Supplementary Figure 3 — Endocytic mediator’s mRNA levels after siRNA injections. Expression levels of Aak1 (A), Dnm1 (B), and Dnm3 (C) mRNA in the spinal cord (SC) and DRG of treated mice, determined by qRT-PCR, n = 4 mice per group. [file Image_3.TIF]
